# Conceiving an application ontology to model patient human papillomavirus vaccine counseling for dialogue management

**DOI:** 10.1186/s12859-019-3193-7

**Published:** 2019-12-23

**Authors:** Muhammad Amith, Kirk Roberts, Cui Tao

**Affiliations:** School of Biomedical Informatics, The University of Texas Health Science Center at Houston, 7000 Fannin Road, Suite 600, Houston, TX, 77030 USA

**Keywords:** Dialogue system, Ontology, Patient provider communication, Conversational agent, Human papillomavirus vaccine

## Abstract

**Background:**

In the United States and parts of the world, the human papillomavirus vaccine uptake is below the prescribed coverage rate for the population. Some research have noted that dialogue that communicates the risks and benefits, as well as patient concerns, can improve the uptake levels. In this paper, we introduce an application ontology for health information dialogue called Patient Health Information Dialogue Ontology for patient-level human papillomavirus vaccine counseling and potentially for any health-related counseling.

**Results:**

The ontology’s class level hierarchy is segmented into 4 basic levels - *Discussion*, *Goal*, *Utterance*, and *Speech Task*. The ontology also defines core low-level utterance interaction for communicating human papillomavirus health information. We discuss the design of the ontology and the execution of the utterance interaction.

**Conclusion:**

With an ontology that represents patient-centric dialogue to communicate health information, we have an application-driven model that formalizes the structure for the communication of health information, and a reusable scaffold that can be integrated for software agents. Our next step will to be develop the software engine that will utilize the ontology and automate the dialogue interaction of a software agent.

## Introduction

The United States is failing to achieve its targeted uptake for human papillomavirus (HPV) vaccination with its low rate of 28% [1], which is a short of the 80% coverage target [2]. If one prediction is correct, 4 millions deaths can be prevented if we were to achieve 70% HPV vaccine coverage [3]. The HPV vaccine is 99% effective in protecting from the HPV virus - a virus that is can lead to life threatening cancers in both men and women in their adult age [4].

In 2014, the President’s Cancer Panel suggested the need for patient-physician counseling for HPV vaccination, and for many patients, their health care provider is their main and most trusted source of health information to learn more about the HPV vaccine [5, 6]. Several studies have all declared that health care provider influence is an important factor to improve HPV vaccine uptake for their patients [7–9, 9–17]. Few studies have noted as high as 95% vaccine acceptance whenever physician counseling occurs between patient and physician, and in one study an 18-fold probability of the acceptance as a result of health provider recommendation occurred [17–23]. Many patients prefer the face- to-face interaction and counseling to learn more about the HPV vaccine in order to decide on vaccine uptake [24, 25]. While there is a strong preference for patient and physician interaction, this unfortunately forces the physician into taking on more intensive health education than time might allow [26]. This would involve additional professional development training and tips to address HPV vaccine barriers in order to effectively communicate health information to patients [21, 27–32]. Also, this would require health care professionals to be aware of myths surrounding HPV vaccine, and detailing facts about HPV and the vaccine, and providing a comfortable atmosphere for patients [25, 28]. There is also the challenge that patients will ask few questions and interact minimally when being counseled on vaccines, as well as the health care provider dominating the discussion and peppering their dialogue with technical jargon that could impede counseling [33, 34]. Yet, health care providers have limited time to discuss the HPV vaccine with patients, which also impacts decisions for vaccination uptake [33]. In addition to the aforementioned points, physicians may also deviate from best practices [35]. Thus, we assume that automating the vaccine counseling session, with conversational agents, may provide a method for standardizing and formalizing dialogue with health consumers, and provide an efficient means to communicate health information that could improve patient satisfaction and patient health literacy.

### Dialogue and Dialogue Systems

A dialogue system, based on the Journal of Dialogue System definition, is

“a computational device or agent that (a) engages in interaction with other human and/or computer participant(s); (b) uses human language in some form such as speech, text, or sign; and (c) typically engages in such interaction across multiple turns or sentences”[36].

Dialogue systems or spoken dialogue systems have the benefit of being:

- enjoyable to use by participants [37]
- ease of usability due to the hands-free nature, and the ability to use natural language commands [38]
- adoption benefit for novice users [38]
- like talking to a real person, even if they were talking a computer [39]

In speech and discourse, one can potentially communicate more information that in the written form [40]. Because speech is a natural act among humans, the ease to express thoughts in speech is relatively easier than in writing [41, 42]. In addition speaking provides opportunity to convey more content in very little time [43–45]. Machines, unlike humans, are not social entities, yet research in text-mining and natural language processing are advancing the possibility of more interactive systems that can help users query systems. With advancements in speech technology and technologies that can support dialogue systems, machines could perform natural conversations and realistically mirror human-to-human interactions in a consistent manner.

Dialogue systems for health care imbues several benefits. In particular, health dialogue systems have the benefit of positively affecting the patient’s health-related behavior and assist in the observing the health-status of the patient. When offered as an alternative to paper-based documentation for patients, automated verbal communication can provide sophisticated goal oriented information delivery for patients [46]. Health dialogue systems with the power of speech recognition can mimic the face-to-face interaction between provider and patient and automate that experience [39]. Specifically, the verbal mode of the health dialogue system can offer opportunities to enhance interactivity between patient and provider, such as using machine intelligence for decision making and coordination of content delivery, utilizing interpersonal cues to imitate human conversation and improve communication efforts with non-experts. It can also personalize the experience with the user [39]. Potentially, a health dialogue system can be cost effective if it is portable and generic, meaning if the system is not coupled with any specific domain [38]. These systems also have the potential to reach a wider audience to deliver health information [47]. Communities that do not speak the native language of the provider can also be positively impacted by health dialogue systems that have multilingual support [48]. While it may not replace the experience between patient and provider, it can help assist both parties - in helping the patient connect with other individuals and promoting self-management of care.

Since the 1990s, health dialogue systems, whether telephone-based or computer-based, have emerged in published medical research and demonstrated usage in a variety of health-based applications. Some examples of health dialogue systems utilization in managed care applications include nutrition[49–51], cigarette [52], hypertension [53], and asthma management [54]. Also health dialogue systems have been demonstrated in health behavioral interventions such as encouraging patients to engage in physical activity [55–57], adhere to medication routines [49, 53, 58], and encourage routine mammography screenings [59].

### Related Studies on Ontology-based Conversational Agents

An ontology is a formalized encoding of knowledge that enables machines and computerized agents to understand domain information. This could enable software agents to perform machine-based reasoning from the semantic logical connections between concepts in the ontology. Ideally, if machines and systems can harness ontologies to understand and reason about domain information, they can possibly wield knowledge about counseling discourse with patients and perhaps lead towards theoretical plan-based dialogue management, which requires reasoning to implement [60].

We reviewed the literature on PubMed for recent work on using ontology-driven solutions for dialogue management for conversation agents, specifically directed to patient-centric counseling. We used the following search query: *ontology AND (dialogue OR conversation OR counseling)*. The query yielded 47 results, and we examined each title and abstract for any relevance to ontology or conversational agents. This reduced our count to 10 papers, which we reviewed. We excluded any papers published before the year 2000 (n=2), and papers that have no relevance to conversational agents and/or ontology-based solution (n=4). We examined 4 papers that had some importance to ontologies and conversational agents.

Beveridge and Fox developed a spoken dialogue system that suggested to physicians on whether patients should be further screened for breast cancer [61]. The system utilized an approach where the dialogue flow is treated as a “game board” that mediates a domain ontology as a knowledge base and domain planner that tracks the state of dialogue. However, according to Bickmore, et al., the system was limited in scale [62]. Also, the system was not a complete ontology-driven solution, as there was only a domain ontology that serves as a knowledge base, and the dialogue flow was handled through XML encoding.

Tielman, et al.’s work involved the use of conversational agents to assist individuals suffering from post-traumatic stress disorder in building 3D virtual worlds to cope with past traumas [63]. The system utilized a set of ontologies to represent information about the location of their trauma. The virtual agent inquired about specifics of their 3D virtual world, and linked the information with the ontology, as the participant was constructing their virtual world.

Finally, two of the papers were by Bickmore, et al., who had developed embodied conversational agents to affect health behaviors for diet interventions [62, 64]. Their work was rooted in utilizing the Transtherotical Model, a behavioral change model that was represented in an ontology and another ontology that coordinated the structure of the utterance exchanges between user and machine as a state-based network. However their work was limited due to scalability to any other domain and would require re-work to adapt. Another limitation was the lack of interoperability of the behavioral change theory ontology and the speech task ontology, which limited any grounding in behavioral theory.

Our overarching goal is to develop a conversational agent with a speech interface for HPV vaccine counseling that is driven by an ontology to coordinate the dialogue system. A study by Miner and colleagues looked at commercially available conversational agents (Alexa, Siri, etc.) to test their feasibility for health-related dialogue interactions and revealed various issues ranging from inadequate and incomplete information, and patient safety issues [65]. In relation to the aforementioned studies, we intend to have the ontology-based dialogue system closely aligned with behavioral theories, and provide a reusable foundation for ontology-based dialogue systems in other domains. The unique potential of using ontologies for conversational agents is the possibility of fusing behavioral change models with dialog to help ground these conversational agents in behavioral change theories [46, 62]. This paper will focus on the core application ontology called the Patient Health Information Dialogue Ontology (PHIDO), inspired from dialogue interaction from a simulated study.

### Overview

The objective of this study is to develop an application ontology for dialogue management in vaccine counseling. Another objective is to create an ontology that is sufficiently generalizable to cover other types of informative discussion on health information. With a general framework, developers for the agents can customize the ontology for any particular domain that involves health-related discussion. In addition, we intend to incorporate a theoretical framework to help ground the discussion and perhaps validate the theory at a later future stage. Particularly with interventions relating to vaccine, theories of health behavior can potentially deduce beliefs that may lead to vaccine uptakes [66].

## Method

**Fig. 1:**
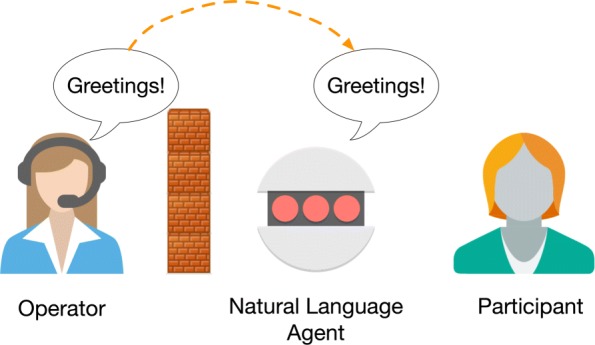
Wizard of OZ experiment with natural language interface agent. “Support Icon” (CC Attribution 3.0) by Squid Ink [97],“Brick wall Icon” (CC Attribution 4.0) by Anna Shlyapnikova [98], “Gnome robots icon” (GNU General Public License v3.0) by Papirus Development Team [99], and “User female alt Icon” (Public Domain license) by paomedia [100]

Our application ontology is based on work and experiences in developing a dialogue script for vaccine counseling which was later executed in a Wizard of OZ experiment conducted from February to July of 2018 [67]. Wizard of OZ protocol simulates dialogue interaction between human and machine (i.e. robot or system natural language interface) [68], and like the story by L. Frank Baum [69], there is a remote operator speaking on behalf of the machine (Fig. 1). This gives the human participant the perception that he or she is conversing to an automated machine and thereby providing their authentic responses. In effect, this will help us test our proposed ontology-based dialogue system in a relatively bug-free process.

The development of the script included refinements from domain experts in public health and from healthcare providers who interact with patients on a daily basis. In addition, the script was framed on the Health Belief Model, using a survey [70] that contains required information about HPV and the HPV vaccine. Based on participant interaction and analysis of the utterances collected during the study, we analyzed the script for potential patterns to be represented as a common framework for communicating health information to patients. Further details of our experiment have been documented [67].

What we observed are three basic types of “tasks” in the simulated interaction - one of which is an exchange of pleasantries, a question and answering task, and other talking point-related task. Within each of these types of speech tasks, we observed several additional sub-types within them. Each of the tasks also contained a set of broad utterance types that had similar sequential flows. In the next sections, we introduce the various representations of the application ontology we called the Patient Health Information Dialogue Ontology (PHIDO). We detailed the Utterance concept and their various subtypes, and the Speech Task concept that utilizes the Utterance concepts to execute the dialogue flow, along with a couple of high level classes (Communication Goals and Discussion) encapsulating the Speech Tasks.

### Utterance Class

**Fig. 2:**
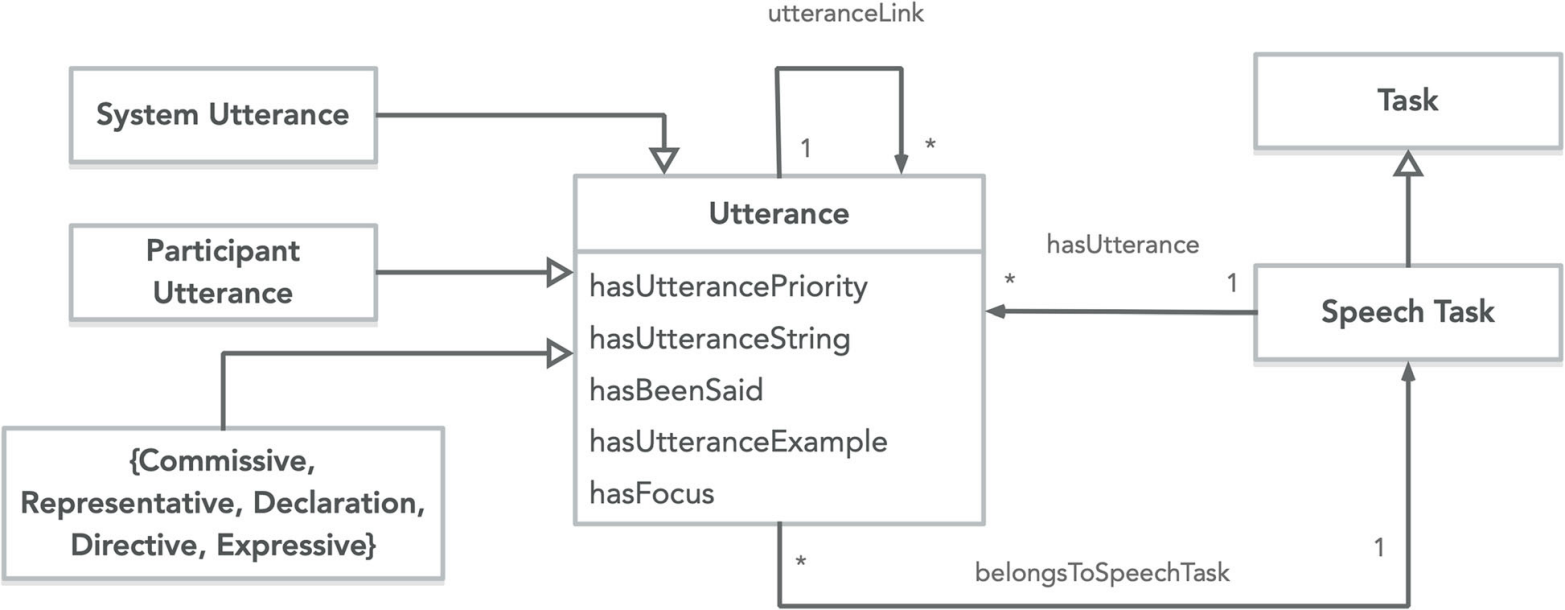
Utterance class concept in UML

The Utterance class (Fig. 2) within the context of the application ontology describes any piece of speech evoked by either the participant user (i.e. the patient or health consumer) or the software agent system (i.e. the conversational agent). Each Utterance concept serializes some data properties - *hasUtterancePriority*, *hasUtteranceString*, *hasBeenSaid*, *hasUtteranceExamples*, and *hasFocus*. The *hasUttereanceString* is a string data type property for the actual piece of text to be spoken by the application system. The *hasBeenSaid* property is boolean type to indicate to the system that this utterance has been evoked before. The *hasUtteranceExamples* is data property for providing the system some semantically similar or keyword texts of the utterance, which could help the application perform some similarity matching to identify the utterance. The *hasUtterancePriority* is integer data type property to denote a priority-based ordering, if the application needs to determine an ordering of utterances. The *hasFocus* is a boolean data property to specify the position of the dialogue flow. This will be later discussed in “Transition mechanism” section where the transition algorithm is outlined.

We incorporated Searle’s classification of speech acts to the Utterance class. Speech acts, also referred to as dialogue acts, are classification of the types of speech based on the function or purpose. Searle introduced his classification of these acts in [71]:

- “Assertive: committing the speaker to something’s being the case (suggesting, putting forward, swearing, boasting, concluding)
- Directives: attempts by the speaker to get the addressee to do something (asking, ordering, requesting, inviting, advising, begging)
- Commissives: committing the speaker to some future course of action (promising, planning, vowing, betting, opposing)
- Expressives: expressing the psychological state of the speaker about a state of affairs (thanking, apologizing, welcoming, deploring)
- Declarations: bringing about a different state of the world by the utterance (including many of the performative examples above; I resign, you’re fired)” [72].

The partial reason for incorporating Searle’s speech classification is to open any opportunity in aligning to an upper level ontology, like BFO (Basic Formal Ontology)[73]. Many well-known biomedical ontologies, specifically those that are domain reference ontologies, are aligned to BFO [74] and BFO contains a specific concept class called “utterance”. Overall, PHIDO is designed as an application ontology which are defined as “an ontology that is created to accomplish some specified local task or application”, contrasting from reference ontologies that are designed to be canonical knowledge of a domain. Any possibility for alignment to BFO would be a downstream prospect.

The Participant Utterance class represents utterances expected and spoken by the user. Table 1 outlines the types of Participant Utterances and the classification associated with Searle’s speech act types. System Utterance represents utterances vocalized by the agent or machine and listed in Table 2 with their Searle’s classification.

**Table 1:** Participant Utterance classes

**Participant Utterance**	**Speech act type**
Acceptance	Expressive
Negative Utterance	
- *Disconfirmation*	Expressive
- *Negative Personal Status*	Representative
Participant Farewell	Expressive
Participant Introduction	Assertive
Personal Status	
- *Negative Personal Status*	Expressive
- *Positive Personal Status*	Expressive
Positive Utterance	
- *Confirmation*	Representative
- *Positive Personal Status*	Expressive
Prattle	*NA*
Question	Directive
- *Divergent Question*	Directive
Reciprocal Farewell	Expressive
Reciprocal Greet	Expressive
Request System	Directive
- *Request System Repeat*	Directive
Unintelligible	*NA*

**Table 2:** System Utterance classes

**System Utterance**	**Speech act type**
Acknowledgment	Expressive
Agenda	Commissive
Answer	Representative
- *No Answer*	Representative
Apology	Expressive
Capitulate	Expressive
Compassionate Utterance	
- *Condolence*	Expressive
- *Happy For*	Expressive
Confirm Health Information	Directive
System Declaration	
- *Disclaimer*	Representative
- *Topic Transition*	Commissive
System Farewell	Expressive
- *Concluding Farewell*	Expressive
System Greet	Expressive
Inform	Representative
Inquire Personal	Directive
Interview Question	Directive
Option	
- *Clarification Options*	Directive
- *Question Options*	Directive
- *Topic Options*	Directive
Overview	Commissive
Request	Directive
Request Repeat	Directive
Satisfaction Prompt	Directive
System Declaration	
- *Disclaimer*	Representative
- *Topic Transition*	Commissive
System Introduction	Representative

Specific classes like Unintelligible or Prattle (Table 1) did not have Searle’s speech act types mainly because there were some utterances that were not captured accurately during the Wizard of OZ experiment. This is comparable to real life human-to-human conversation, where a hearer may not completely discern what is being said by the speaker, or if the speaker is uttering speech that may be deemed as drivel or nonsensical.

**Fig. 3:**
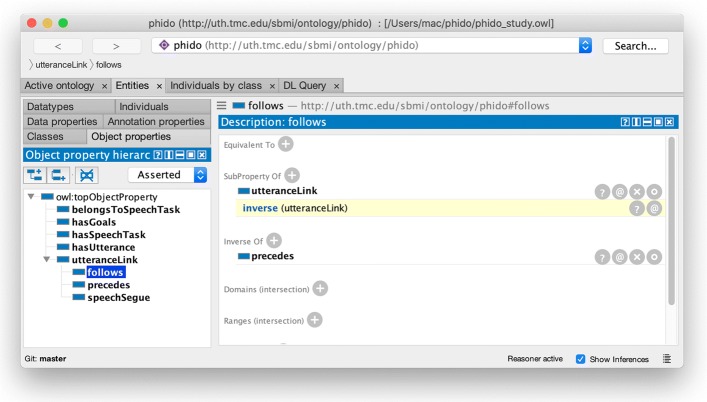
Screenshot of Protégé showing the follows data property with an iverse of the precedes data property

Object data properties were defined between the Utterance and the Speech Task. Each Utterance class has a prospective link to a Speech Task, *belongsToSpeechTask*, and in reverse, *hasUtterance* defines an object property link from Speech Task to Utterance class. Lastly, each Utterance is linked with the object property of *utteranceLink*. The utteranceLink was defined as symmetrical object property and is sub-classed by *follows* and *precedes* (Fig. 3), both of which were encoded as inverse of each other. So if *Utterance*_*a*_>*precedes*>*Utterance*_*b*_, then *Utterance*_*b*_>*follows*>*Utterance*_*a*_. *speechSegue* is another sub-property of *utteranceLink* used as a transition to link the last Utterance of a Speech Task to the next Speech Task.

### Task Class

**Fig. 4:**
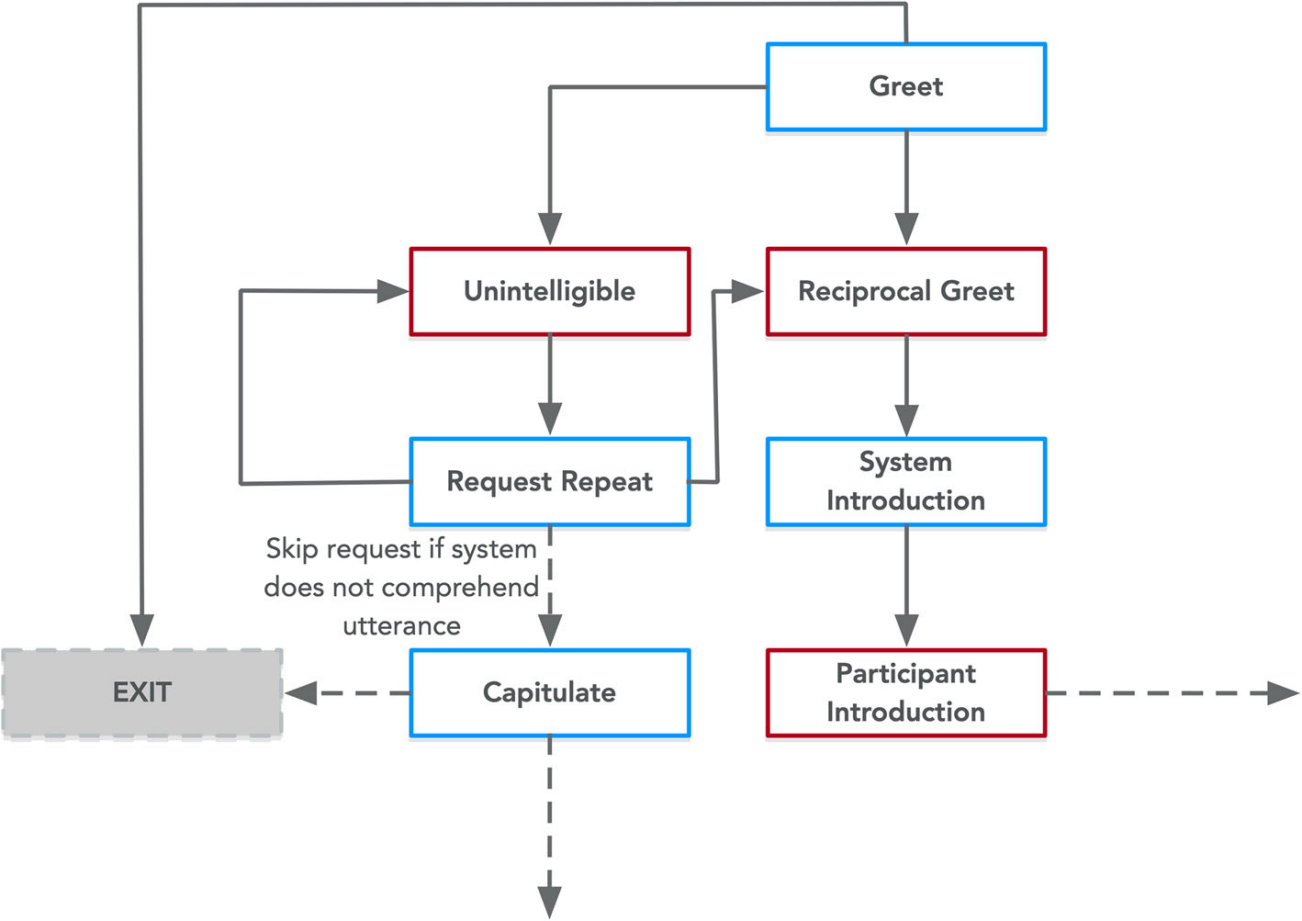
Expression of the Salutation class from PHIDO. Blue is the System Utterance and red is the Participant Utterance

**Fig. 5:**
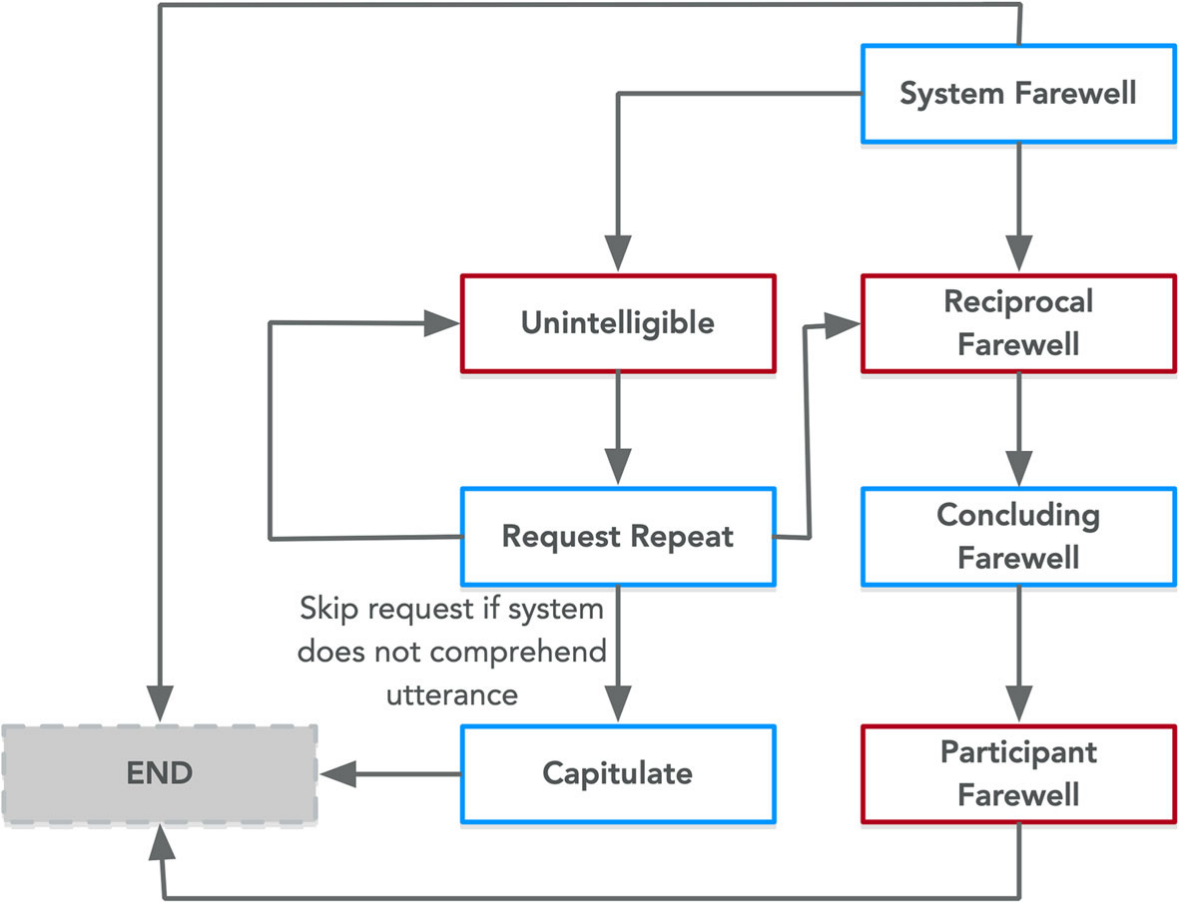
Expression of the Valediction class from PHIDO using a set of utterance classes. Blue is the System Utterance and red is the Participant Utterance

**Fig. 6:**
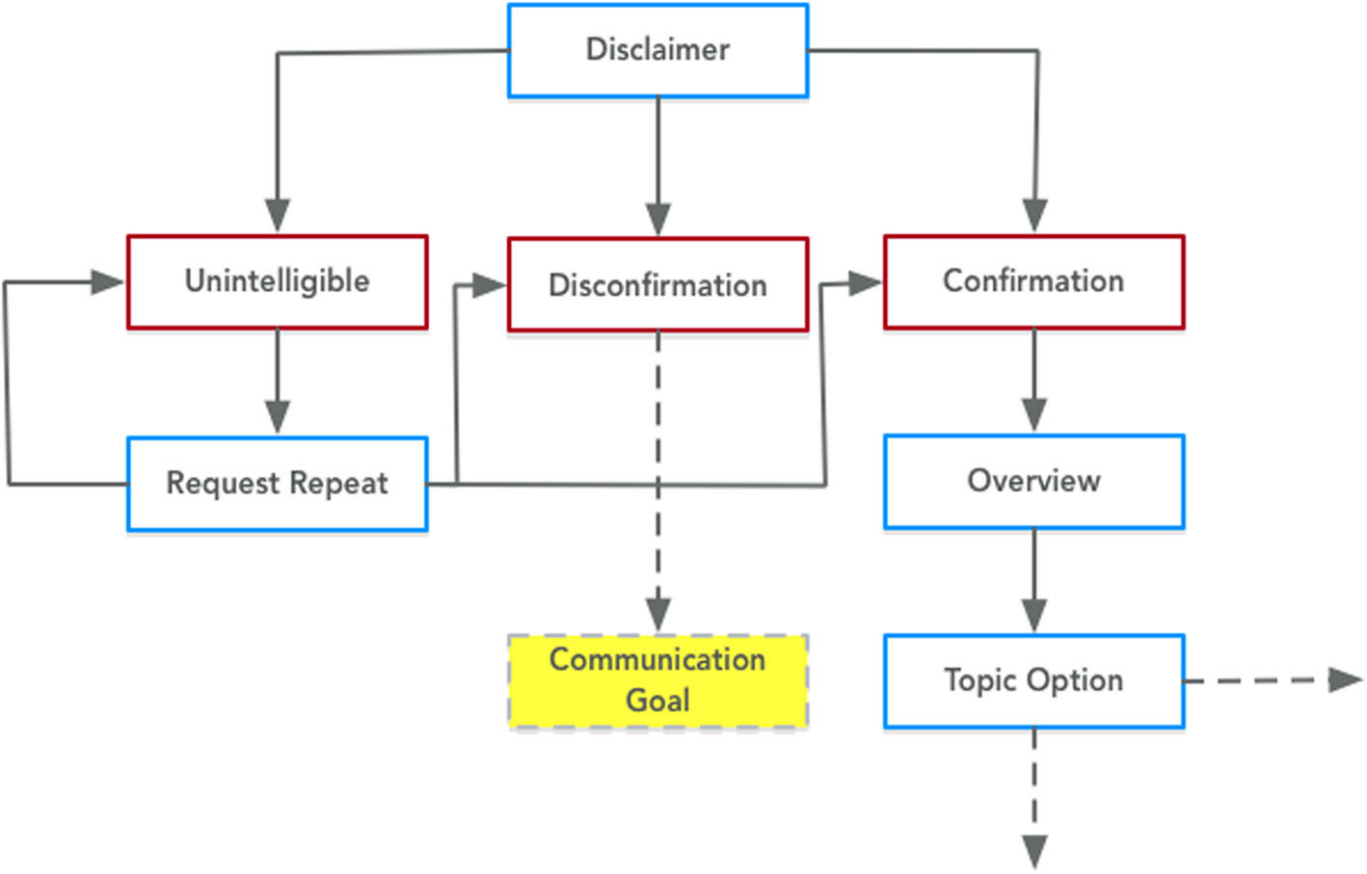
Expression of the Initiate Discussion class from PHIDO using a set of utterance classes. Blue is the System Utterance and red is the Participant Utterance

**Fig. 7:**
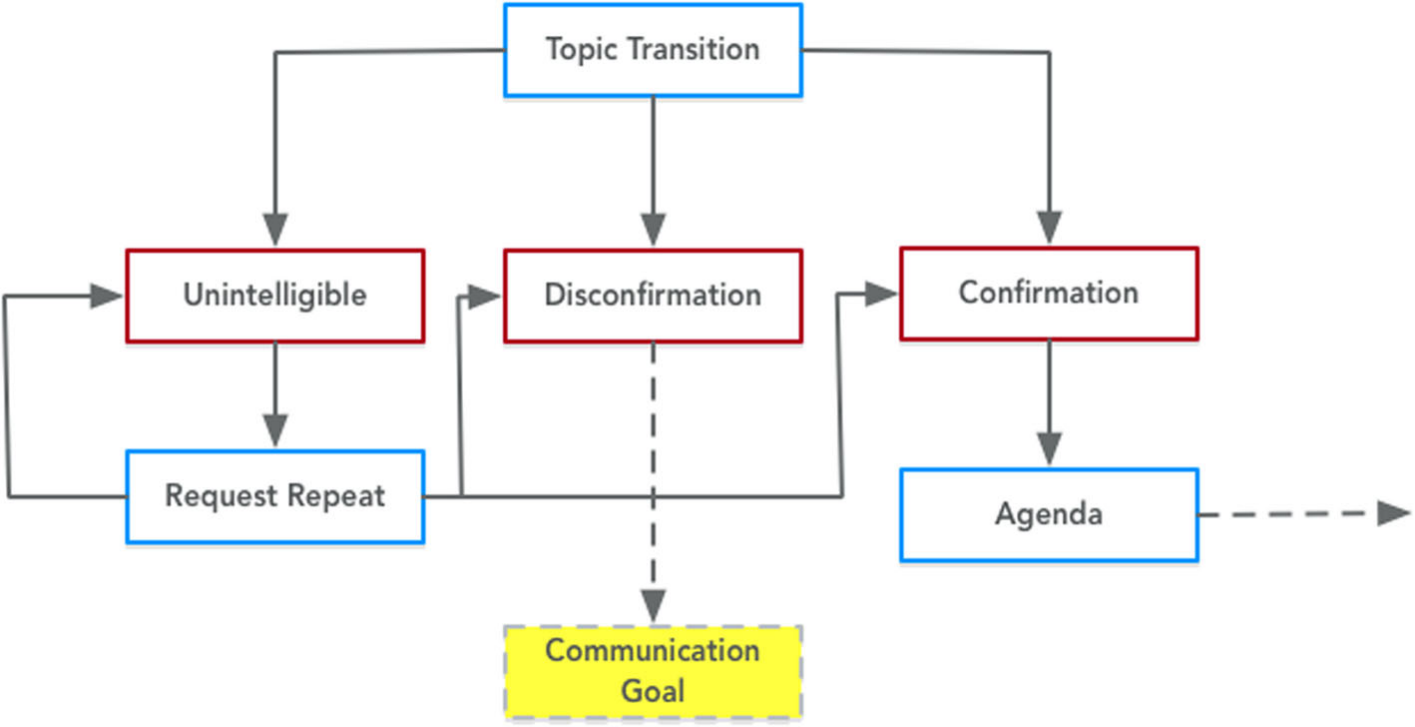
Expression of the Transition to Topic class from PHIDO using a set of utterance classes. Blue is the System Utterance and red is the Participant Utterance

**Fig. 8:**
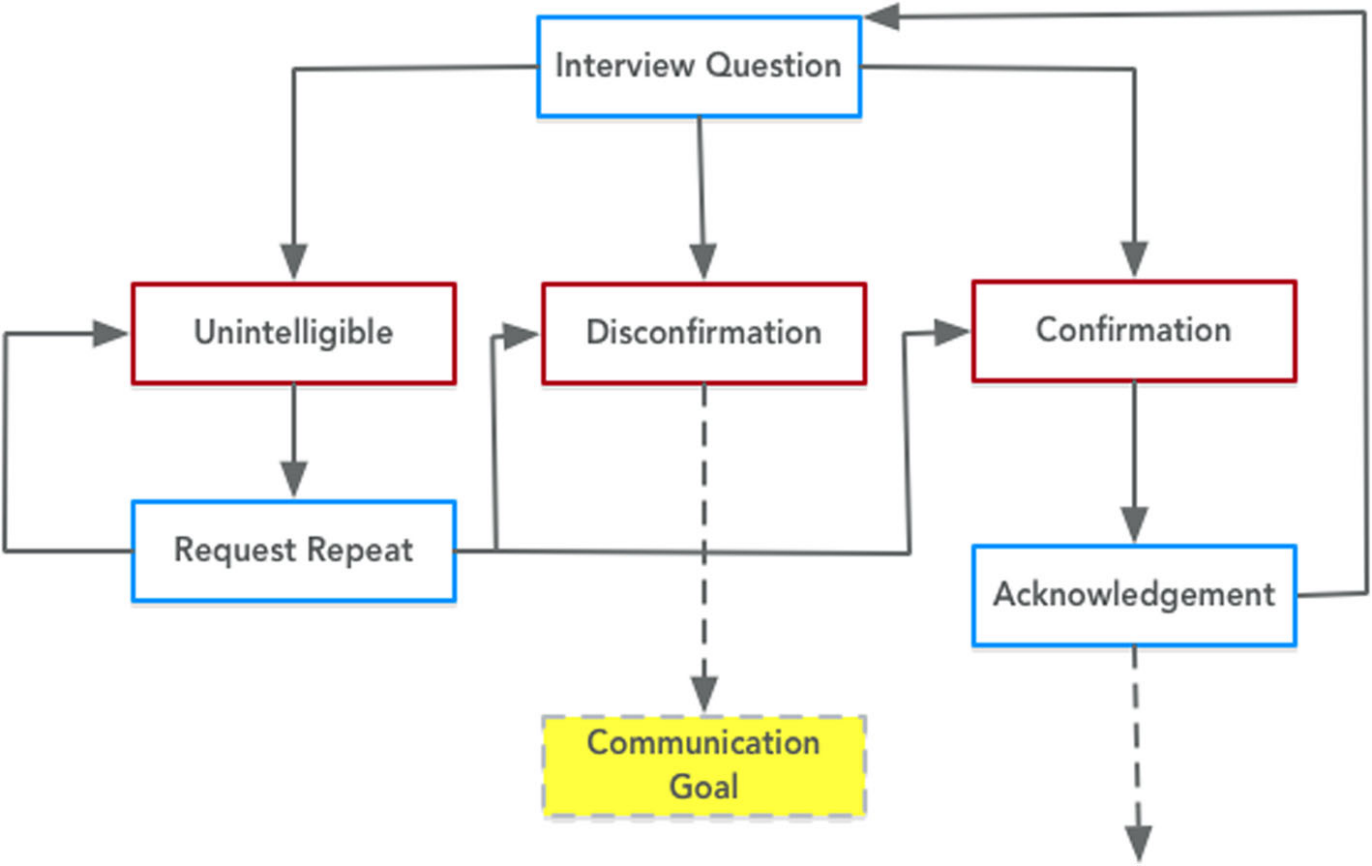
Expression of the Interview Participant class from PHIDO using a set of utterance classes. Blue is the System Utterance and red is the Participant Utterance

**Fig. 9:**
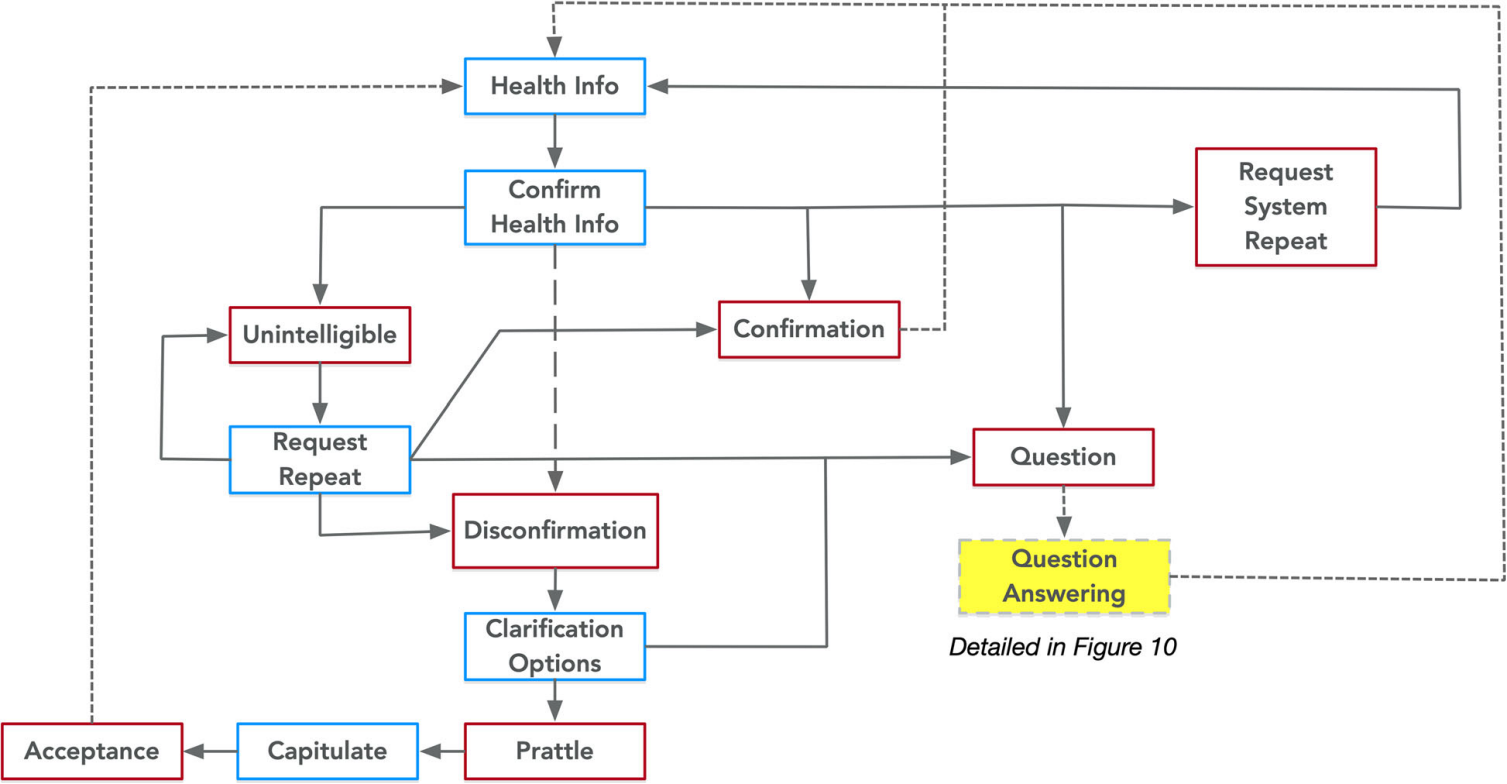
Expression of the Discuss Health Topic class from PHIDO using a set of utterance classes. Blue is the System Utterance and red is the Participant Utterance

**Fig. 10:**
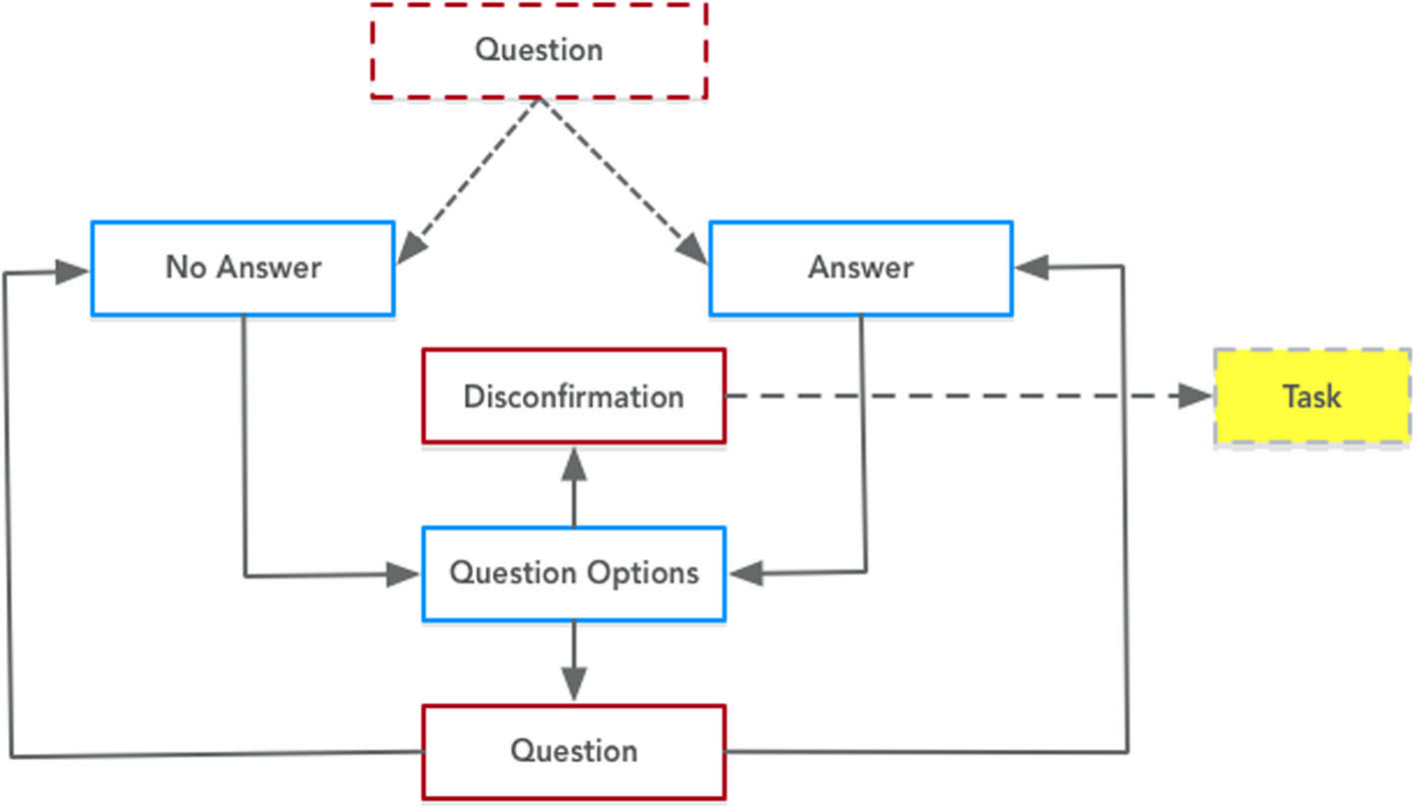
Expression of the Question Answering Task class from PHIDO using a set of utterance classes. Blue is the System Utterance and red is the Participant Utterance

We defined Speech Task as a type of Task, a generic concept to represent activities. Speech Task was designed to execute a specific atomic objective involving a series of utterances (Utterance). Earlier we noted several types of Speech Task - one involving the exchange of pleasantries (Pleasantry Task), another involving communicating ideas (Proposition Task), and a question and answering activity (Question and Answering). Figures 4- 5, 6, 7, 8, 9 and 10 represent the variations of the Speech Task.

#### Pleasantry Task

The Pleasantry Task models simple introductory and concluding portions of the counseling session from the Wizard of OZ experiment to simulate some human-like formality with the participant user.

The introductory portion is called the Salutation Task (Fig. 4) which starts off with a greeting by the system and then followed by expected utterances of either a returned greeting by the participant (Reciprocal Greet) or the possibility of the system receiving an utterance that might be gibberish (Unintelligible). If the latter, Salutation Task involved a request for repeat. If the system using the ontology continues to misunderstand the utterance, the ontology leads the system to end the counseling session. Otherwise, the system will continue as modeled.

The concluding portion called the Valediction Task is similar in its sequence with the Salutation Task except for the types of utterances utilized (i.e. System Farewell instead of Greet, Concluding Farewell instead of System Introduction, etc.). Figure 5 shows the Valediction Task as modeled in the PHIDO ontology.

#### Proposition Task

The Proposition Task is a subclass of Speech Task, and this concept pertains to communicating a piece of information with some feedback where the participant user acknowledges the information he/she hears. One variation of the Proposition Task modeled the introduction of the overall theme of the discussion (Initiate Discussion), and other variations of the Proposition Task included probing the participant’s information (Interview Participant), switching to a different topic (Transition to Topic), and communicating a health fact (Discuss Health Topic). For most of the Proposition Tasks, there is a sequence to handle repeat of utterances (Request Repeat), or if the participant user has any contentious responses (Disconfirmation >*speechSeque*>Communication Goal) that could lead to a set of dialogue sequence to nudge the user back into the counseling session or address concerns.

Figure 6 displays the Initiate Discussion task that aims to introduce the theme of the counseling session. The ontology directs the application with any disclaimer (Disclaimer); in the case of HPV vaccination, our Wizard of OZ sessions declared to the participant that the agent may not accommodate all of the concerns or cover the spectrum of knowledge on the HPV vaccine, but it advised that the participant should seek out their provider for any unanswered questions or personal concerns. The PHIDO ontology facilitates the response to the disclaimer and pilots the dialogue into an overview of the discussion (Overview) and then prompts for topics that might initially interest the participant (Topic Option).

The Interview Participant (Fig. 8) was designed to be a basic speech activity to gain some insight about the participant user. For example, in some dialogue system approaches like mixed initiative, dialogue systems inquire about contextual information about the user, and with the information, the system can automate a customized conversation with the user. Another example is small talk with the user for the purpose of acclimating the user to the agent (i.e. "How is your day going, Alice?"). Interview Participant, like all other Proposition Task have the same utterance sequence, but starts off by asking a question (Interview Question) which then directs the flow of the conversation to where the system recognizes or captures the information about the user (Acknowledgment). Potentially, the ontology can loop back to asking another question or moving to the next Speech Task.

The crucial part of the PHIDO ontology for communicating health information to the participant user is the Discuss Health Topic (Fig. 9). The goals of this task is to confirm that the user understands the information that is communicated, answer any relevant or tangential questions that the user may have, and address any concerns about the communicated health information. In the sequence of this Speech Task type, one piece of information (e.g. “HPV vaccine might cause some minor discomfort and pain or soreness at the injection site.”) is spoken by the agent (Health Information) and later the agent inquires if the user understands this (Confirm Health Information). From here, similar to other Proposition Tasks, the ontology helps the application to handle the expected utterance of the participant user - repeating of information (Request System Repeat), clarify the user’s utterance (Unintelligible >*precedes*>Request Repeat), or attend to user’s issue with the information (Disconfirmation). Discuss Health Topic incorporated a question and answering transition if the participant user has a follow up question (Question). After the iteration, the ontology manages the flow of the dialogue to the next instance of Discuss Health Topic.

### Question and Answering Task

The original dialogue script permitted users to ask questions during the counseling session, and during the Wizard of OZ experiment, several users asked questions. This is reflected in the previously mentioned Speech Tasks after a piece of information was communicated (Fig. 9).

Figure 10 shows the utterance sequence for question and answering encoded into PHIDO. From the Question in Discuss Health Topic, the agent provides a response that either answers the participant user question (Answer) or a default non-answer (No Answer). Afterwards, the agent prompts the user if he/she has more questions (Question Option) or if they should proceed (Discomfirmation >*precedes*>Speech Task).

### Discussion and Goal Class

**Fig. 11:**
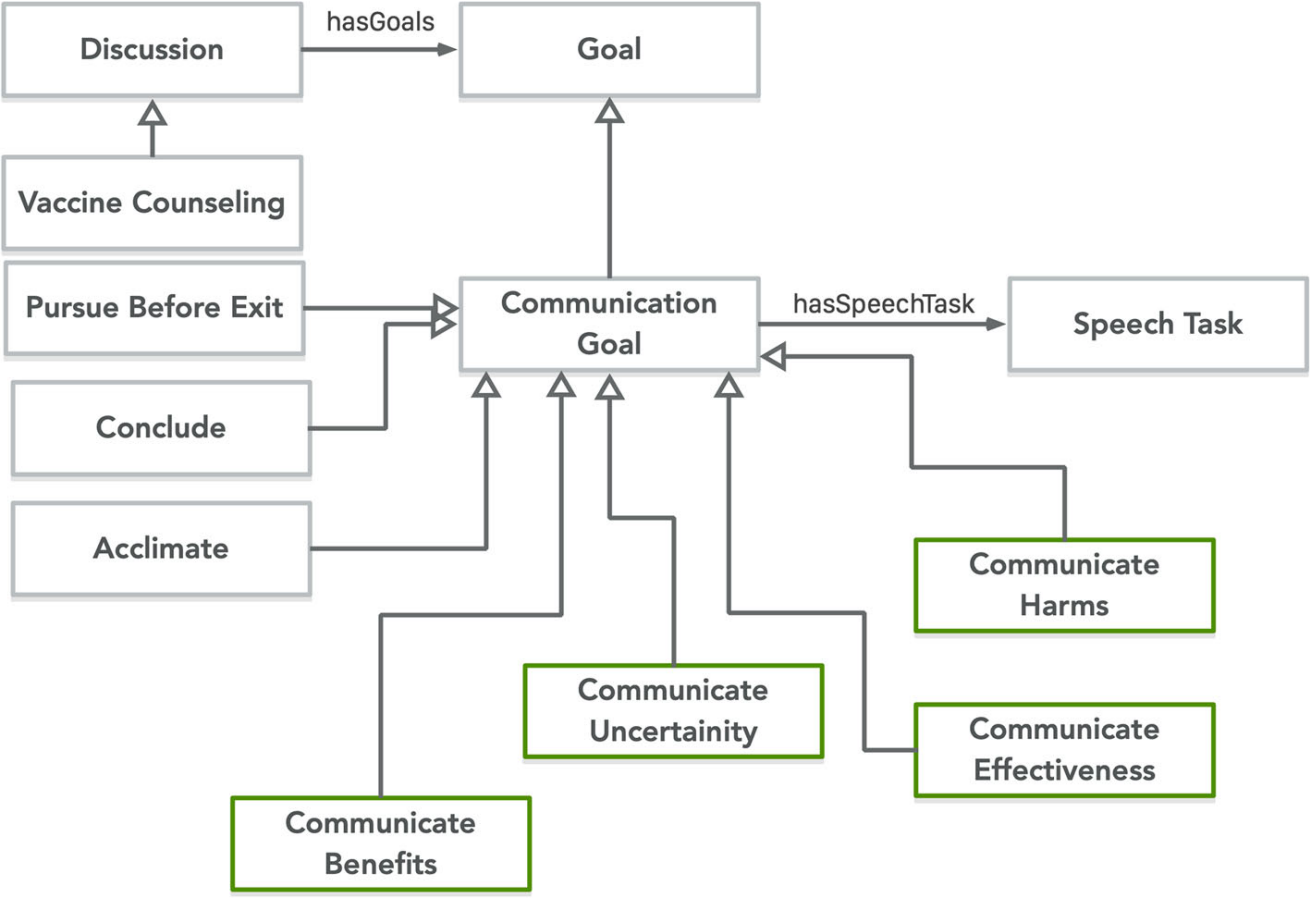
Communication Goal and Discussion concept represented within PHIDO. Green concepts are related to the Health Belief Model constructs

PHIDO provided classes to structure the Speech Tasks and Utterances (Fig. 11). Vaccine Counseling class is a subclass of Discussion, and it aimed to encapsulate the entire counseling experience with *hasGoal* relationship to the Goal concept. The Communication Goal is a subclass of Goal that has an object property to link various Speech Tasks (*hasSpeechTask*). Specific to vaccine counseling, we defined some high level abstract communication goals that were reflected in dialogue script and the simulated counseling session with participants of the Wizard of OZ protocol. One Communication Goal was Acclimate that was designed to help the user adapt to the experience of talking to an automated conversational agent. While the Acclimate goal introduced the user to a conversational agent and the agenda of the discussion, we also defined a Conclude as another Communication Goal for ending the discussion.

Four other Communication Goals were Communicate Benefit, Communicate Effectiveness, Communicate Harms, and Communicate Uncertainty. These four Communication Goals were based on the Health Belief Model constructs, a behavior model that leads to change in action, specifically uptake to vaccine. In HPV vaccine research, studies have revealed that the Health Belief Model has shown to predict the intent to take the HPV vaccine among young females [75, 76], and many other studies have utilized the Health Belief Model to determine subject’s intent for vaccine uptake, specifically the HPV and the influenza vaccine [77–80]. Most of the talking points were reversed engineered from the Carolina Health Belief Model Survey, a categorical Health Belief Model survey [70]. So each talking point was related to their specific categorical construct (Benefits, Effectiveness, Harms and Uncertainty). In addition, we represented a Communication Goal called Pursuit Before Exit. This was based on research by Opal and colleagues that explored the use of presumptive nudging and tone to encourage patients, who may be hesitant, to adhere to vaccination [81]. Part of their research involved the use of “pursuing” the patient if they simply reject the adhering to vaccination schedule.

## Results and Discussion

We encoded PHIDO using Protege and serialized the ontology in the Web Ontology Language (OWL2) [82]. PHIDO contains 86 classes and 14 properties (9 object and 5 data). PHIDO does not have any instance level data. Instance level data is reserved for the utterances and application data when PHIDO is integrated with a software agent. The current iteration of PHIDO is available at https://bitbucket.org/tuanamith/phido.

### Ontology Metrics

For an initial quality-based assessment of PHIDO, we utilized a semiotic metric suite introduced by Burton-Jones and colleagues that measures an ontology based on the branches of semiotic theory (*semantic*, *syntactic*, and *pragmatic*) [83]. Each of the scores in the metric range from 0 to 1, and the composite of the scores provided an overall score – ((0.33·*syntactic*) + (0.33·*pragmatic*) + (0.33·*semantic*)). In a previous study, we generated scores for a BioPortal sample to serve as comparison benchmark to assess drug ontologies [84]. We used this benchmark comparison data to compare with PHIDO’s metrics to ascertain its quality with other ontologies. To calculate PHIDO, we imported the ontology to OntoKeeper, a prototype tool we developed that facilitates the aforementioned semiotic metric suite [84]. OntoKeeper is powered by OWL-API [85] and other natural language processing libraries to parse and calculate the data from the ontology.

PHIDO’s *syntactic* score, which measure the quality of syntax language of the ontology, was 0.69. The sub-scores for *syntactic*, *lawfulness* and *richness*, were 1 and 0.38, respectively. *Lawfulness* indicates any syntactic violations to OWL2 profile. The high score of 1.00 reveals no syntactic violations. *Richness* highlights the percentage amount of unique types of logical axiom ontology features. The score of 0.38 revealed that PHIDO only used about a third of these features. In comparison, with the Bioportal sample, the z-score for *syntactic* score was z=0.36 (z=0.5 for *lawfulness* and z=0.11 for *richness*) indicating a better syntactic-level quality.

*Semantic* score measured an ontology’s quality of term labels. The *semantic* score for PHIDO was 0.94. The *semantic* score comprises of *interpretability*, *consistency*, and *clarity*. *Interpretability* measured at 0.94, *consistency* measured at 1.00, and *clarity* was 0.92. Z-score for *semantic* score rated at z=0.40 (z=0.43 for *interpretability*, z=0.40 for *consistency*, and z=-0.31 for *clarity*). While the overall *semantic* score was better, the sub-score for *clarity* was low compared to the National Center for Biomedical Ontology (NCBO) sample’s *clarity* sub-score. This may indicate that the term labels have some ambiguity (i.e. term labels that has above average number of word senses).

The *pragmatic* score assessed the ontology’s domain coverage and utilization. This score was limited to its sub-score of *comprehensiveness*. The other sub-scores of *pragmatic* included *relevance* and *accuracy* which required external assessment resources (domain experts). Essentially, *comprehensiveness* measured the ontology’s domain coverage based on its size in comparison with the average size of a ontology library. The *pragmatic* score (*comprehensiveness*) when rounded to nearest two digits was 0.00. Z-score yielded z=-0.29, below average in domain coverage than the average NCBO ontology, which indicates that the ontology may need to be further expanded (e.g. more Speech Tasks to be modeled).

The *overall score* for PHIDO is a mean value of the previously mentioned scores (formula goes here) to indicate general assessment of the ontology. PHIDO’s *overall score* was 0.54 and when compared, the z-score value was z=0.43. The *overall score* indicated that PHIDO was above average quality compared to most published ontologies, however, as noted, the domain coverage and ambiguity of term labels were lacking.

### Transition mechanism

**Fig. 12:**
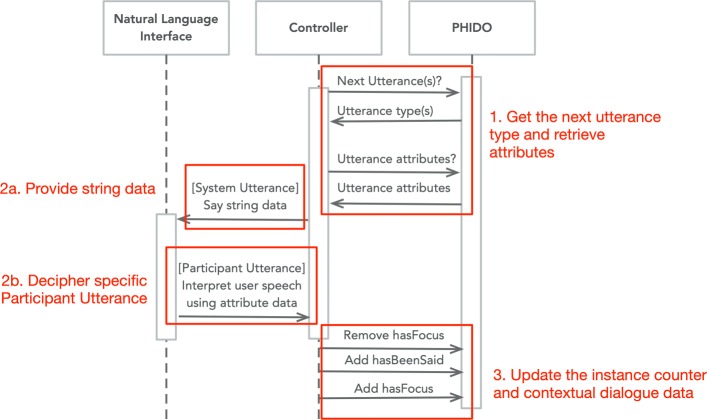
UML sequence diagram describing transition sequence of PHIDO

Earlier, we described some object properties that link the utterances together. The purpose of those links is to implement the application ontology’s ability to guide the software agent to transition from one utterance to another. Essentially, the actual utterance and the system data (Communication Goals and Speech Tasks) are to be serialized as instances in PHIDO. Each class in PHIDO has a *hasFocus* data property attribute that tells the software agent if an instance of that class is current (i.e. where the conversation is at). Overall, a software controller will interact with the Utterance and Speech Task instances to facilitate the movement of the dialogue flow (Fig. 12).

In general, the implementation of the transition starts with a query asking what type of Utterance(s) follows the current instance of the Utterance (*hasFocus*). The system record the following Utterance instance and its attributes, and then the system “kicks back” the Utterance type along with the attribute data (e.g. *hasUtteranceString*, *hasUtteranceExample*, etc.) to the software controller. If the Utterance type is a Participant Utterance, the system will be responsible in discerning the user’s speech with the type of Participant Utterance (e.g. Request System Repeat, Question, etc.). If the Utterance type is a System Utterance, the system will be responsible for using the natural language interface to say the string from the *hasUtteranceString*. Once the system identifies the specific Utterance and preforms a function based on the Utterance, the software controller will respond to the ontology by removing the *hasFocus* instance data and insert a new instance of *hasFocus* data property to the following Utterance instance.

### Limitations and Future Directions

The representation of the patient-centric counseling was inspired from the dialogue script and the Wizard of OZ implementation of the script with live participants, which included the interaction logs from their participation. While the application ontology is rooted in real world activity, there will likely be exceptional utterances that we may not anticipate by future user interactions. Currently, our Wizard of OZ experiment is ongoing and future interaction logs may inspire modifications for the ontology.

We stressed that PHIDO is an application ontology, so it may not universally cover the domain of dialogue interaction, nor are there immediate plans to align it with an upper ontology. Currently our focus is on vaccine counseling, but we foresee the possibility that PHIDO could cover patient-centric communication of health information for a variety of topics while being grounded in some behavioral theory. Also of similar importance, as indicated by the domain coverage, PHIDO would need to model additional Speech Tasks since it only represented 6 types, which is not extensive.

Dialogue management is bifurcated into dialogue flow and dialogue context components [60]. Most of what we discussed for PHIDO facilitates dialogue flow with some minimal contextual information (e.g. Utterance class’s *hasBeenSaid*). Ideally, we hope in the future that PHIDO will encompass management for dialogue flow and contextual information for an ontology-driven approach.

Currently, we are developing a software wrapper, called COO (Conversational Ontology Operator), that will implement the transition algorithm. This dialogue engine is a software library that uses RDF4j and OWL-API that automates the dialogue managements tasks directed by the PHIDO and the aforementioned transition mechanism. Using the utterance data from the dialogue script and chat logs collected from our Wizard of OZ experiments we intend to populate the ontology with instance-level data and test it with randomized selections from user utterances. Also, we plan on reporting a qualitative assessment with the Trindi tick-list [86], a survey for dialogue systems, with prospective users.

#### Ontology-based Question and Answering System

Question answering (QA) is “the task of finding answers to natural language questions, meaning that question answering systems do not retrieve documents (like information retrieval systems), but instead provide short, relevant answers in an interactive setting” [87]. Essentially, the aims of QA is to help users use natural language to find precise information and help end-users query knowledge sources without having to code computer-level queries, using a natural language interface [88].

There are benefits for an ontology-driven method for question answering over other question answering methods [89]. For the last decade, several ontology-driven QA tools were proposed - AquaLog [90], PANTO [91], NLPReduce [92], Freya [93], Querix [94] - with relative success. While they each introduced their various approaches, they all exhibit some similar features. This included a gazetteer subsystem that build a list of terms utilized in the ontology, along with some procedures to preform term similarity between terms from the query and the gazetteer. Additionally, another similar feature among the QA systems is a process to extract knowledge triples from the natural language query, facilitated by a natural language parser or a combination of a few natural language methods.

We also plan on embedding an ontology-based question-answering system to handle participant users’ questions. Our future approach will incorporate new developments in natural language processing and ontology research, and introduce some experimental approaches to improve retrieval of knowledge encoded in an ontological knowledge base. Our previous work included developing a patient-centric vaccine knowledge base (VISO [95] and VISO-HPV [96]) which will be used as the “brain” of the conversational agent to answer the user’s questions. Our future research aims may offer a lightweight method that is suitable for small devices and contribute to the body of research to "talk to the semantic web".

## Conclusion

We derived an application ontology for dialogue management called Patient Health Information Dialogue Ontology (PHIDO) that is based on our on-going Wizard of OZ experiments conducted at University of Texas Health Science Center. This application ontology is intended to be used in a prospective dialogue engine for embedded and mobile devices that will automate a counseling session for HPV vaccine, a vaccine that has dramatically low coverage among the population. Our initial qualitative results based on semiotic metric suite indicated that PHIDO is of comparable quality to NCBO Bioportal ontologies. Our current activity is to develop the software engine that will harness PHIDO to be deployed in machines, and to link a lightweight ontology-based question and answering system to the dialogue manager. We foresee that our work will demonstrate and contribute to the usefulness of semantic web and ontology technology to power patient-centric conversation for health information.

## Data Availability

Not applicable.

## Abbreviations

BFO: Basic formal ontology
COO: Conversational ontology operator
HPV: Human papillomavirus
NCBO: National center for biomedical ontology
OWL2: Web ontology language
PHIDO: Patient health information dialogue ontology
QA: Question answering
